# Chasing the thermodynamical noise limit in whispering-gallery-mode resonators for ultrastable laser frequency stabilization

**DOI:** 10.1038/s41467-017-00021-9

**Published:** 2017-03-31

**Authors:** Jinkang Lim, Anatoliy A. Savchenkov, Elijah Dale, Wei Liang, Danny Eliyahu, Vladimir Ilchenko, Andrey B. Matsko, Lute Maleki, Chee Wei Wong

**Affiliations:** 1 Mesoscopic Optics and Quantum Electronics Laboratory, University of California, Los Angeles, CA 90095 USA; 2 OEwaves Inc., 465 North Halstead Street, Suite 140, Pasadena, CA 91107 USA

## Abstract

Ultrastable high-spectral-purity lasers have served as the cornerstone behind optical atomic clocks, quantum measurements, precision optical microwave generation, high-resolution optical spectroscopy, and sensing. Hertz-level lasers stabilized to high-finesse Fabry-Pérot cavities are typically used for these studies, which are large and fragile and remain laboratory instruments. There is a clear demand for rugged miniaturized lasers with stabilities comparable to those of bulk lasers. Over the past decade, ultrahigh-*Q* optical whispering-gallery-mode resonators have served as a platform for low-noise microlasers but have not yet reached the stabilities defined by their fundamental noise. Here, we show the noise characteristics of whispering-gallery-mode resonators and demonstrate a resonator-stabilized laser at this limit by compensating the intrinsic thermal expansion, allowing a sub-25 Hz linewidth and a 32 Hz Allan deviation. We also reveal the environmental sensitivities of the resonator at the thermodynamical noise limit and long-term frequency drifts governed by random-walk-noise statistics.

## Abstract (web summary)

High-quality optical resonators have the potential to provide a miniaturized frequency reference for metrology and sensing but they often lack stability. Here, Lim *et al*. experimentally characterize the stability of whispering-gallery resonators at their fundamental noise limits.

## Introduction

High-precision optical frequency metrology, spectroscopy^1, 2^, atomic clocks^3–5^, optical interferometry^6^, ultralow phase noise microwave generation^7^, and light detection and ranging^8^ benefit from stable and spectrally pure laser oscillators. Such low-frequency noise oscillators can be achieved by stabilizing laser oscillators to a high-quality factor cavity resonance (*Q* = *ν*/Δ*ν*, where *ν* is the resonance frequency and Δ*ν* is the full-width at half maximum of resonance). When the signal-to-noise ratio (SNR) of the detected laser signal is high enough in the measurement bandwidth (BW), the frequency stability can also be improved, which is scaled by (*Q* × SNR_BW_)^−1^. The benchmark ultrahigh-*Q* resonances in nature are atomic transitions. The precise transitions of trapped atoms have been utilized for improving stability and frequency noise of both microwave and optical oscillators. For instance, the cesium hyperfine transition is the core building block of the well-developed microwave atomic clock and the optical transitions of trapped neutral and ionic atoms are used for modern optical atomic clocks. Although a laser with frequency instability of 6 × 10^−16^ for 2–8 s integration time has been demonstrated in a cryostat via spectral-hole burning written in the Eu^3+^:Y_2_SiO_2_ absorption spectrum^9^, making small atomic traps is demanding such that the development of solid-state compact optical references is of immense interest. Laser oscillators with high-finesse optical Fabry-Pérot **(**FP) cavities have been demonstrated with sub-Hz linewidths and fractional frequency instabilities at the 10^−15^ levels in 1–10 s integration time, which is the noise limit imposed by the unavoidable thermal motion of the cavity’s reflection multilayer coating, by either using ultra-low-expansion material in vacuum or temperature cooling to operate the cavity at the zero thermal expansion point in a cryostat^10–15^. However, direct miniaturization of such FP mirror cavities to a microscale size is challenging due to the quality of the cavity mirrors such that their applications have largely remained in the laboratory environment.

Over the last decade, ultrahigh-*Q* whispering-gallery-mode (WGM) resonators^16, 17^ have been implemented for developing low-noise microlasers and microclocks^18–21^, which show the broad transparent window and ultrahigh-*Q* resonances without sophisticated dielectric mirror coatings and are tolerant to mechanical noise. Since the early studies on the fundamental thermal fluctuations in microspheres by^22^, theoretical predictions indicate that the thermodynamically bounded frequency instability of the WGM resonators can be better than 10^−13^ in 1 s integration time^23, 24^ if the proper material and stabilization technique are selected. However, in spite of the tremendous progress, the existing WGM resonators still experience large frequency instabilities and long-term frequency drifts^25–28^ hindering their use in precision metrology and timing applications.

Here, we show the noise characteristics of a WGM resonator at the fundamental thermodynamical noise limit. We employ a thermal-compensation design for reducing the thermal sensitivity of a conventional WGM resonator and reveal the residual environmental sensitivities of the compensated resonator. Subsequently, we suppress the environmental perturbations using an evacuated rigid enclosure and demonstrate that a laser stabilized to the thermal-compensation WGM resonator, working as an optical probe, shows a spectral linewidth of <25 Hz and a fractional frequency instability of 1.67 × 10^−13^ (5.0 × 10^−12^) on the 191 THz carrier at 0.1 (1) s integration time, which is the best among the WGM resonators of the given size and morphology without a stringent ambient temperature control. Furthermore, we confirm that the centre-shifted random walk noise statistics, imposed by the correlation between the WGM resonator temperature change and the ambient temperature or pressure variation, triggers the long-term frequency instability and monotonic frequency drift of the laser stabilized to the resonator at the thermodynamical noise limit.

## Results

### Thermal-compensation ultrahigh-*Q* WGM resonator

**Fig. 1:**
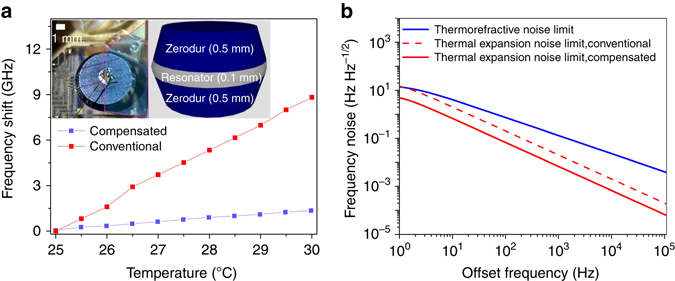
Thermal-compensation MgF_2_ resonator. **a** Measurement of the frequency shifts for the conventional MgF_2_ WGM resonator (*red*) and thermal-compensation WGM resonator (*blue*), respectively. *Inset*: The MgF_2_ resonator (radius: 3.45 mm and thickness: 0.1 mm) sandwiched by Zerodur layers (thickness: 0.5 mm each) in the packaged unit. The MgF_2_ resonator (*middle*) is glued in between two Zerodur components (*top* and *bottom*). The thickness of Zerodur layers is determined by the thermo-mechanical properties of the resonator. **b** Thermorefractive noise limit (*blue*) and thermal expansion noise limit (*red*) of the WGM resonator. The simulations show that the thermal expansion noise of the compensated resonator (*solid red line*) is lower than the thermorefractive noise near the carrier frequency while the conventional one (*dashed red line*) is similar. Therefore, the fundamental thermorefractive noise fluctuation can be clearly reached with the compensated resonator in this experiment

The thermal-expansion coefficient of the crystalline MgF_2_ is ~9 ppm K^−1^, that is a large value compared with the conventional reference FP cavities possessing <0.1 ppm K^−1^. To reduce the thermal sensitivity causing the thermo-mechanical fluctuations via expansion of the resonator length, we compensate the thermal sensitivity of a thin MgF_2_ WGM resonator. The thermal compensation, sandwiching a WGM resonator with laminated Zerodur as illustrated in the inset of Fig. 1a, is guided by numerical simulations and predicts the significant reduction of the thermal sensitivity (Supplementary Note 1 and Supplementary Fig. 1). The design is applied to a MgF_2_ WGM resonator with a 6.9 mm diameter and 100 (25) μm resonator (rim) thickness. The thickness of the Zerodur layer (up and down) used for the MgF_2_ WGM resonator is 500 μm. To find the degree of thermal compensation, we take a pair of WGM resonators and mount them on the same temperature-stabilized platform. While two low-noise continuous wave (cw) lasers are locked to the WGM resonances, respectively, the temperature of one of the WGM resonators is slowly changed and the relative frequency shift of the beatnote between the two stabilized lasers is measured. We assume that all slow changes in the beat frequency are attributed to the thermal expansion of the WGM resonator because the thermorefractive coefficient of crystalline MgF_2_ is significantly smaller than the thermal expansion coefficient. The measurement in Fig. 1a shows ~7 times improvement compared with a conventional MgF_2_ WGM resonator. Although the enhancement factor is smaller than the value predicted by numerical simulations, attributed to the residual thermal expansion due to the imperfection in the device fabrication, the measurement confirms the validity of our thermal-compensation design. The compensated WGM resonator is packaged into a small form factor (40 × 40 × 15 mm^3^) with an integrated thermal sensor and a Peltier-type thermoelectric cooler under the WGM resonator as well as a prism coupler and a photodetector. Laser light is delivered to the prism coupler using a polarization-maintaining single mode fiber with a firmly mounted output tip. The temperature of the WGM resonator is stabilized at 301.2 K by a proportional-integral-derivative (PID) feedback control using the thermoelectric cooler. We then measure the thermal sensitivity again by measuring the beat frequency between a cw laser stabilized to the WGM resonator and a cw laser referenced to the ultrastable FP cavity (Stable Laser Systems) possessing 1 Hz linewidth and 0.1 Hz s^−1^ drift-rate while the set-temperature of the PID control changes, resulting in 3 ppm K^−1^. From the measured thermal sensitivity, we calculate the noise limits imposed by the thermo refractive and thermal-expansion sensitivities of the resonator (Supplementary Note 2), which are the two dominant thermal noise sources for WGM resonators, as illustrated in Fig. 1b. After the thermal compensation, the thermal-expansion noise limit is lower than the thermorefractive noise limit near the carrier frequency as shown in Fig. 1b (and Supplementary Fig. 2) such that the thermorefractive noise-limited fluctuations can be unveiled. The WGM resonator has the unloaded resonance BW of 26 kHz and the loaded resonator *Q* is characterized with 4 μs ring-down time corresponding to 2.4 × 10^9^ at 191 THz carrier (Supplementary Note 2 and Supplementary Fig. 2).

### Resonant frequency shift due to ambient perturbations

**Fig. 2:**
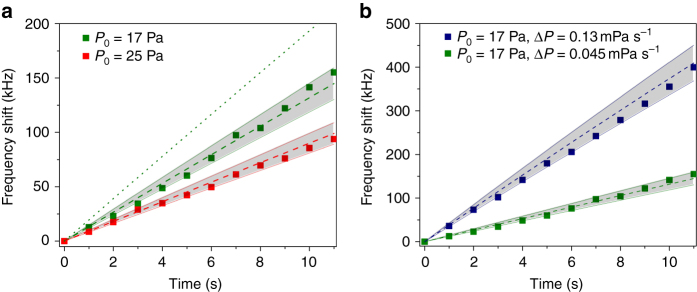
Pressure dependence of the WGM resonance frequency shift. **a** The pressure in the vacuum chamber is slowly increased from 17 Pa (*olive*) and 25 Pa (*red*) with 45 mPa s^−1^. The frequency shift is measured by counting the beat frequency at every second while the laser is stabilized to the WGM resonator by Pound-Drever-Hall locking. To confirm the frequency shift by the pressure change, the theoretically estimated lines from Eq. (1) for TM mode (*dashed line*) and Eq. (2) for TE mode (*dotted line*) are plotted together. The measurement values agree with the theoretical model within the 10% error bars agree with the theoretical model. For comparison, the 10% error zones of the theoretical values are added (*gray*). **b** The pressure in the vacuum chamber is increased from 17 Pa with 45 mPa s^−1^ (*olive*) and 130 mPa s^−1^ (*navy*), respectively. The measurements also agree with the theory within the 10% error zone of the theoretical values

The mechanism behind the resonant frequency shift of a WGM resonator by the ambient perturbation is the interaction between the evanescent wave of the WGM resonator and the air refractive index change caused by temperature and pressure variations. The temperature and pressure are, in principle, coupled quantities connected by the ideal gas law (*PV* *=* *nRT*) in a rigid box, where *P* is the pressure, *V* is the volume, *n* is the number of moles, *R* is the ideal gas constant, and *T* is the absolute temperature. Therefore, the ambient temperature stability required for achieving the thermodynamical noise limited fluctuation can be derived from the pressure stability measurement. For this measurement we place the thermal-compensation WGM resonator in a rigid vacuum chamber on vibration isolation pads. A 3 kHz laser operating at 191 THz is stabilized to the compensated WGM resonator using the Pound-Drever-Hall (PDH) locking technique^29^ that provides sufficient technical noise suppression and thus enforces the laser frequency to chase the resonance (Methods; Supplementary Note 3 and Supplementary Fig. 3). The resonant frequency shifts induced by pressure changes are measured by counting the beat frequency between the stabilized laser and the FP reference laser at every second. Figure 2a shows the laser frequency shift when the pressure slowly increases (Δ*P* = 45 mPa s^−1^) in the vacuum chamber from *P*_0_ = 17 and 25 Pa, respectively. At the given pressure *P*_0_ and the change (Δ*P*), we measure 13 (9) kHz with *P*_0_ = 17 (25) Pa for a transverse magnetic (TM) mode input. Here, we define that the TM mode of our WGM resonator has the electric field distribution primarily in the radial direction. We also measure the resonant frequency shift at the different pressure increment. While the pressure in the vacuum chamber is increased from 17 Pa with Δ*P* = 130 mPa s^−1^, we measure the frequency shift of 36 kHz (Fig. 2b), which shows approximately a linear relationship between the frequency shift and the speed of pressure change. To confirm our measurements, we quantify the impact of the refractive index change of the surrounding air medium on the WGM resonant frequency shift for TM and transverse electric (TE) modes using equations derived from the first order perturbation theory in ref. 30.

1 $$\frac{\Delta {\nu }_{{\rm{TM}}}}{{\nu }_{0}}=-\frac{\Delta {n}_{{\rm{air}}}}{{({n}_{{\rm{TM}}}^{2}-1)}^{3/2}}\frac{\lambda }{2\pi r},$$

2 $$\frac{\Delta {\nu }_{{\rm{TE}}}}{{\nu }_{0}}=-\frac{\Delta {n}_{{\rm{air}}}}{{({n}_{{\rm{TE}}}^{2}-1)}^{3/2}}(2-\frac{1}{{n}_{{\rm{TE}}}^{2}})\frac{\lambda }{2\pi r},$$

3 $$\Delta {n}_{{\rm{air}}}={n}_{0}|\Delta P/{P}_{0}|,$$

where *r* is the radius of the WGM resonator, *n*_TE_ and *n*_TM_ are the refractive indices of the resonator host material, *λ* is the wavelength, Δ*n*_air_ is the change of the refractive index of air and *n*_0_ is the residual refractive index of air given by the air itself, that is ~ 3 × 10^−4^, and therefore, Δ*n*_air_ ≈ 7.94 × 10^−7^ in these measurements. The calculated results from Eq. 1 for *P*_0_ = 17 (25) Pa are Δ*ν*_TM_ = 13.3 (9.1) kHz at Δ*P* = 45 mPa s^−1^, which agrees well with our measured values of 13 (9) kHz for a TM mode input as illustrated in Fig. 2a. The theoretical frequency shift from thermorefractive noise of our WGM resonator is estimated by <(Δ*ω*_tre_)^2^> = *ω* ^2^(*α*_n_ *T*)^2^ *k*_B_ (*CV*_m_ *ρ*)^−1^ ≈ (2*π* 46 Hz)^2^, where *α*_n_ is the thermorefractive coefficient, *T* is the cavity temperature, *k*_B_ is the Boltzmann constant, *C* is the specific heat capacity, *V*_m_ is the mode volume, and *ρ* is the density of the host material. The values used for the theoretical estimates are given in Supplementary Note 2. Theoretical estimations and experimental measurements indicate that the pressure change (Δ*P*) has to be controlled under 0.154 mPa to unveil the fundamental thermorefractive noise limit of the compensated WGM resonator. Using the ideal gas law, we calculate the corresponding temperature variation of 2.7 mK, in which *n* *=* 3.53 × 10^−6^ mole, *V* *=* 0.52 L, and *R* = 0.082 atm L mol^−1^ K^−1^ are used. This measurement shows that pressure and temperature of the surrounding air have to be controlled better than those of open atmospheric environments to approach the thermodynamical noise limit of the WGM resonator.

The pressure variation not only changes the ambient air refractive index but could also deform the resonator. Therefore, we consider the resonant frequency shift due to the change in the ambient pressure at the given volume of the WGM resonator leading to a frequency shift by

4 $$\frac{\Delta {\nu }_{{\rm{TM}}}}{{\nu }_{0}}=\frac{1}{3}{\beta }_{{\rm{T}}}\Delta P,$$

Where *β*_T_ = −[(1/*V*)(*δV*/*δP*)]_T_ is the compressibility of the resonator host material (we assume that isothermal and adiabatic compressibility are approximately equal). The compressibility of MgF_2_ is *β*_T_ = 10^−11^ Pa^−1^ and, therefore, the frequency shift due to the pressure change (Δ*P*)of 45 mPa is 29 Hz, which is insignificant in this measurement.

### Frequency noise spectrum of the thermal-compensation WGM resonator

**Fig. 3:**
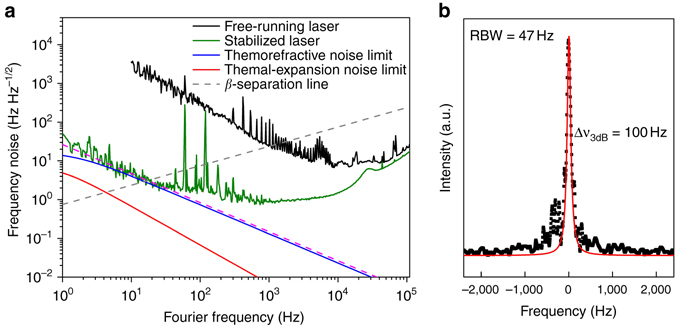
Frequency noise of the thermal-compensation resonator. **a** The frequency noise power spectral density (FNPSD) measured by the beat frequency between the laser and the table top Fabry-Pérot (FP) cavity reference laser. The FNPSD of free-running laser used in our experiment (*black curve*) and of the laser stabilized to the compensated resonator (*olive*). The thermodynamical FNPSD predictions from thermorefractive (*blue*) and thermal expansion noise (*red*) are also illustrated for comparison. The thermorefractive noise shows *f* ^−1.5^ frequency dependence (*magenta dashed line*) predicted by our theoretical model. **b** The measured linewidth (fullwidth at half maximum) on a spectrum analyzer with 47 Hz resolution bandwidth (RBW) and 40 ms sweep time over the 5 kHz span, giving a ≈ 100 Hz Lorentzian linewidth fit. The effective linewidth evaluated from the *olive curve* is 119 ± 2 Hz and the linewidth without the 60 and 120 Hz power-line noise is determined to be 25 ± 0.3 Hz

To minimize the impact of the technical noise from our laboratory environment, the vacuum chamber is evacuated to the pressure of 1.33 mPa but the chamber temperature is not controlled (see also Supplementary Fig. 4). Figure 3a shows the comparative frequency noise power spectral density (FNPSD) curves of the laser stabilized to the thermal-compensation WGM resonator. For comparison, the theoretically estimated thermodynamical noise limit of the WGM resonator is also plotted. The FNPSD of the free-running laser is shown by the black curve and the noise is substantially suppressed (30 dB at 10 Hz) as shown by the olive curve when the laser is stabilized to the WGM resonator. Below 30 Hz offset frequency, the stabilized laser FNPSD curve falls off as *f* ^−1.5^ implying the thermorefractive noise limit of many thermal modes of the WGM resonator predicted by Matsko *et al*.^23^ (Supplementary Note 4). From 30 to 100 Hz, the FNPSD curve falls off with *f* ^−1^ implying the impact of flicker noise caused by the residual laser noise and electronic device noise. Two strong peaks originate from 60 Hz harmonics of the electrical power-line noise. The rising frequency noise above 1 kHz in the *olive curve* is due to the poles in the active feedback loops.

The highest spectral purity laser is achieved among the WGM resonators of the given size and morphology via the thermal compensation. The integral linewidth is evaluated from the FNPSD and turns out to be 119 Hz but when the two 60 Hz harmonic peaks are removed, it is <25 Hz (Supplementary Note 5). To further support our linewidth estimations, we measure the beat signal linewidth (Fig. 3b) in a spectrum analyzer with a resolution BW of 47 Hz and sweep time of 40 ms over the 5 kHz span. Due to the slow frequency drift, the measured line shape shows the asymmetry in the wings but the peak center is nearly symmetric and can be fitted with a Lorentzian lineshape. The resulting full-width at half-maximum linewidth is approximately 100 Hz, matched with the FNPSD measurement including the 60 Hz noise harmonic peaks.

### Frequency stability of the thermal-compensation WGM resonator

**Fig. 4:**
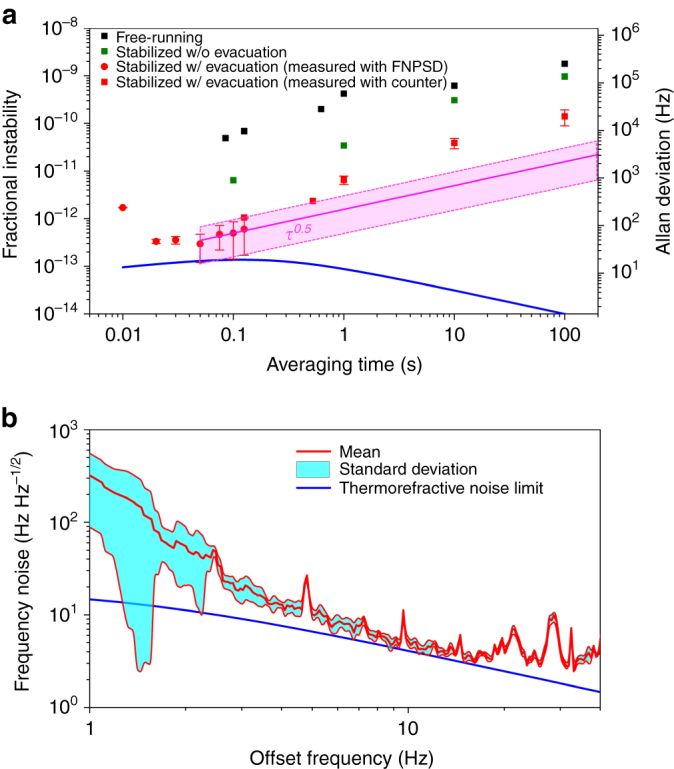
Fractional frequency instability measurements. **a** Measured fractional frequency instabilities (FI) of the beat frequency between the Fabry-Pérot (FP) cavity reference laser and the free-running laser (*squares in black*), and the beat frequency between the reference laser and the laser stabilized to the WGM resonator without evacuation (*squares in olive*), respectively. The *red squares* and *bars* are the mean $(\mu ={n}^{-1}\sum _{i=1}^{n}{{\rm{FI}}}_{I})$ and standard deviation $(\sigma =\sqrt{{n}^{-1}\sum _{i=1}^{n}{({{\rm{FI}}}_{I}-\mu )}^{2}})$ of the frequency instability of the laser stabilized to the resonator calculated from 10 sets of frequency counting data at 1 s averaging time, respectively, measured with a frequency counter where *n* is the number data set. The *red circles* and *bars* are *μ* and *σ* of the frequency instability of the laser stabilized to the resonator derived from the 74 frequency noise power spectral density (FNPSD) measurement traces. The *magenta* area represents the expected Allan deviation bound imposed by random walk frequency noise. **b** The noise statistics of 74 FNPSD traces. The *red curve* is the mean value and the *cyan area* is the connected standard deviations. The *blue line* is the thermorefractive noise limit. Both mean frequency noise and standard deviation are reduced along the offset frequency and the frequency noise reaches the thermorefractive noise limit at 6 Hz. However, the thermo-mechanical noise, induced by the residual thermal expansion, is still concentrated near the carrier causing the standard deviation of the Allan deviation measurements in **a**

The resonant frequency instability of the compensated WGM resonator is analyzed by its Allan deviation. We use the FNPSD measurements to evaluate Allan deviations below 0.1 s averaging time because the frequency error of our counter (Agilent 53131 A) increases at smaller than 0.1 s averaging times due to the increasing dead time (Allan deviation at 0.1 s averaging time calculated from the FNPSD curve is compared with the values measured by the frequency counter—both are within the error bars of each other). Figure 4a shows the measured Allan deviations. We plot the Allan deviations from 0.01 to 0.1 s averaging time by statistically evaluating 74 measurement traces of FNPSD (circles in red). The lowest measurement has an Allan deviation of 32 Hz at 0.1 s averaging time, which agrees with the frequency instability imposed by the theoretically estimated limit from the thermorefractive noise of the WGM resonator. We observe the noise floor at 500 μs averaging time and the Allan deviations show a *τ* ^0.5^ dependence (magenta line) along the averaging time implying random walk frequency noise, and thus it is different from the theoretically calculated thermorefractive-noise limit (blue curve) excluding the random walk noise statistics. The statistically estimated Allan deviation at 0.1 s averaging time has a mean value of 95 Hz and a standard deviation of 68 Hz. To understand this deviation, we analyze the mean and standard deviation of 74 FNPSD measurement traces in the low Fourier frequency regime (1–40 Hz) shown in Fig. 4b. The red line is the mean value and the cyan area is the connected standard deviations along the offset frequency. Both mean frequency noise and standard deviation are diminished along the offset frequency until 20 Hz and reaches the thermorefractive noise limit. However, the residual thermo-mechanical fluctuations still exist near the carrier and change in each measurement causing the standard deviation in Allan deviation measurements. This measurement shows that the WGM resonator has relatively high noise sensitivity at low Fourier offset frequencies and, therefore, further reduction of the thermal sensitivity via the thermal compensation is desirable to reduce the measurement uncertainty.

### Random walk noise distribution and long-term frequency instability

**Fig. 5:**
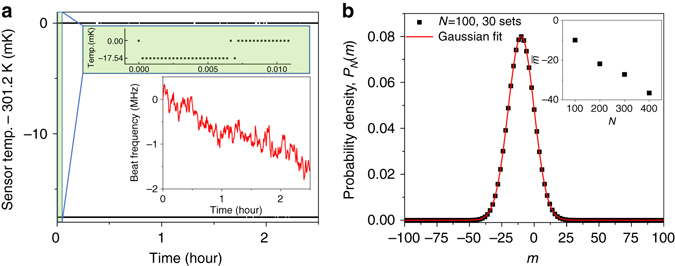
Thermistor sensor temperature random walk distribution statistics. **a** The 10 kΩ thermistor sensor temperature measurement data. Due to the temperature digitization, the measurement resolution is limited such that the *upper* and *lower* temperature bounds per every one second are recorded. *Inset*: resonance frequency shift during the temperature measurement period. **b** The probability distribution for *N* = 100, *P*_*N=*100_ *(m)*, where $\bar{m}$ is located at −10.05 and $\overline{{(\Delta m)}^{2}}$ is 9.97, which is approximately $\sqrt{N}$ demonstrating that this measurement follows the random walk distribution. The *inset* shows that the increased number of samples monotonically shifts $\bar{m}$ far from the centre

The beat frequency at 1 s averaging time is recorded by a frequency counter and the Allan deviations at the longer averaging time are calculated by averaging the 1 s measurement data sets. To check the reproducibility of the stability measurements, we take 10 measurement sets at 1 s averaging time for ~10 min each and analyze them statistically as illustrated in Fig. 4a. The Allan deviations (squares in red) along the averaging time start to deviate from the *τ* ^0.5^curve, which implies a frequency drift. To understand this behavior, we record the temperature of the 10 kΩ thermistor sensor used for detecting the WGM resonator temperature for two and a half hours while the laser is stabilized to the WGM resonator (Fig. 5a). The temperature data is statistically analyzed as shown in Fig. 5b. Due to the digitization of the measured temperature with 10 kΩ thermistor, the resolution is limited such that we can only measure the upper and lower temperature bounds. The inset of Fig. 5a illustrates that the laser frequency shift is <2 MHz during this measurement period and, therefore, the WGM resonator temperature is actually stabilized with <10 mK instability when the measured thermal-expansion sensitivity of 3 ppm K^−1^ is considered. To illuminate the monotonic frequency drift, we apply the random walk and binomial distribution for 30 sets of the number of upper (*n*_u_) and lower bound (*n*_d_) temperature data points with the number of samples (*N*) from the continuously measured temperature data set (Supplementary Note 6 and Supplementary Table 1). The different numbers of *N* are chosen and the average positions ($\bar{m}$) and the standard deviations ($\overline{{(\Delta m)}^{2}}$) are calculated for each case. Then we calculate the probability distribution (*P*_*N*_ *(m)*) defined by

5 $${P}_{N}(m)=\frac{N!}{[(N+m)/2]![(N-m)/2]!}{p}^{(N+m)/2}{q}^{(N-m)/2},$$

where *m* *=* *n*_*u*_ *–n*_*d*_ *=* *2n*_*u*_ *–N, p (q)* is the probability that the measured temperature point is at the upper (lower) bound, respectively, and they satisfy the relation, *p* + *q* *=* 1. From the measurement data, we deduce the values of *p* and *q* and they are 0.45 and 0.55, respectively. Figure 5b shows the probability distribution for *N* = 100, *P*_*N=*100_ *(m)*. The average position, $\bar{m}$ is not at the centre but at −10.05 and the standard deviation, $\overline{{(\Delta m)}^{2}}$ is 9.97 that is approximately $\sqrt{N}$ demonstrating that this measurement follows the random walk distribution. By increasing *N*, the average position shifts monotonically far away from the centre in Fig. 5b inset implying that the measured temperature is monotonically shifted to one of the temperature bounds along the increasing integration time, leading to the frequency drift. This frequency shift could be mitigated by improving the resolution of temperature sensing that is currently limited by the 10 kΩ thermistor sensor, because the narrower bound of the measured temperature reduces the range of the frequency drift. This could be realized by implementing the dual-mode temperature compensation technique to the WGM resonator^27, 28^ allowing a detection sensitivity of 100 nK. We also note that the monotonic long-term frequency drift shows a correlation with the monotonic ambient temperature change inferring that the ambient temperature change triggers the feedback to compensate the WGM resonator temperature accordingly. Therefore, an ambient temperature-controlled enclosure might be necessary to enhance the long-term stability.

## Discussion

We have shown that the impact of the surrounding medium perturbations is a major problem when the WGM resonator stability approaches the thermodynamical fluctuation limit. The ambient temperature stability to reach the thermorefractive noise limit of our thermal-compensation WGM resonator is 2.7 mK and, therefore, the environment temperature control is necessary to enhance the frequency stability. An evacuated environment provided such temperature stability in the short integration time such that the thermal-compensation WGM resonator showed the *f* ^−1.5^ frequency noise dependence imposed by thermorefractive noise of the resonator, and allowed the laser line width of <25 Hz and the lowest Allan deviation of 32 Hz in 100 ms integration time, corresponding to the frequency instability of 1.67 × 10^−13^ for the 191 THz carrier. The standard deviation of the Allan deviation measurements is attributed to the thermo-mechanical noise concentrated near the carrier originating from the residual thermal expansion noise, which suggests that the enhanced thermal compensation is desirable to improve the stability. In principle, it is possible to achieve an order of magnitude less thermal-expansion sensitivity, which could even more alleviate the required ambient temperature stability. In the longer integration time, the laser stabilized to the WGM resonator experiences the monotonic frequency drift due to the centre-shifted random walk probability distribution of the WGM resonator temperature, which is attributed to the limited temperature sensing accuracy and the correlation of the WGM resonator temperature change with the ambient temperature variation. Hence, we anticipate that the long-term frequency drift could be mitigated by implementing the dual-mode temperature compensation technique and by tightly controlling the environmental temperature. Finally, it is noteworthy that ultrahigh-*Q* WGM resonators provide enhanced nonlinearity permitting generation of optical frequency combs with low input power, and, therefore, stable microcombs excluding external references are potentially possible via a single WGM resonator, which could advance the microcomb system in size-, weight-, and power-constrained environments.

## Methods

### Laser stabilization to the thermal-compensation WGM resonator

The 15 mW output power from the self-injection locked laser diode is split into two paths by a 50/50 splitter after an acousto-optic modulator (AOM). One arm is used for stabilizing the laser and the other is reserved for characterizing the noise of the resonator by heterodyne-beating the stabilized laser against a 1 Hz FP cavity reference laser. The piezoelectric transducer (PZT) attached to the laser is used to control the laser frequency. The PZT can be controlled either by a digitized signal on computer interface or by an analog voltage input for frequency modulation to obtain the beat frequency within the photodetector BW (New Focus model 1611). The beatnote is usually generated between 1 and 1.5 GHz and is down-mixed to 50–100 MHz for counting the beat frequency. We place an AOM before the WGM resonator, which assists the frequency stabilization and advances noise suppression in the acoustic offset frequency regime by extending the feedback BW (Supplementary Note 3). We apply the PDH locking technique to stabilize the laser to the WGM resonator. The laser light (1 mW) is phase-modulated by a fiber-coupled electro-optic modulator (5.5 dB loss) and then launched into the resonator. The transmitted light is detected by an internal photodetector in the packaged aluminum box and the detected signal is demodulated with the same microwave source that phase-modulates the laser light at a double balanced frequency mixer (model:ZAD-1+), which produces an error signal. The error signal is optimized by choosing the optimum modulation frequency and the light intensity into the resonator, which are typically ~ 12 MHz and ~ 20 μW, respectively. The error signal is split into two branches and fed into commercial high-speed proportional-integral servo controllers (New Focus, LB1005). A slow servo branch acts on the laser PZT and a fast servo branch is used to further suppress acoustic and laser technical noises. The feedback signal from the fast servo controller is fed into a voltage controlled oscillator (VCO). The VCO output is amplified and then applied to the AOM, which shifts the laser frequency to control the frequency noise up to 400 kHz. The contribution of each servo loop is optimized to achieve the lowest noise level.

### Data availability

The data that support the plots within this paper and other findings of this research are available from the corresponding author on reasonable request.

## Electronic supplementary material

Supplementary Information Supplementary Notes, Supplementary Figures, Supplementary Table and Supplementary References
