# Supplementary material for: Chasing the thermodynamical noise limit in whispering-gallery-mode resonators for ultrastable laser frequency stabilization
Source: Nat Commun. 2017 Mar 31;8:8. doi: 10.1038/s41467-017-00021-9 (PMC5431900; doi:10.1038/s41467-017-00021-9)
Supplement: Supplementary file 1 — Supplementary Notes, Supplementary Figures, Supplementary Table and Supplementary References [file 41467_2017_21_MOESM1_ESM.pdf]

Supplementary Note 1. Device fabrication

To compensate for the extensive thermal expansion of a whispering-gallery-mode (WGM) resonator, the resonator is sandwiched by a nearly zero or slightly negative expansion material (e.g. Zerodur). The compensating layers should be thick enough to hold the resonator's expansion. The ambient temperature variations should be small (< 5 ^{o}C) to avoid resonator destruction. The layers are bonded together with a transparent glue and then annealed with slightly varying temperature.

We determined the thickness of the compensating layer on the resonator to fully suppress an overall frequency shift for a given resonator's diameter and thickness via COMSOL simulations (Supplementary Figure 1a). The glue layer has 2 μm thickness and is taken into account in the numerical simulation. The colour code indicates the linear expansion of the WGM resonator's equator. The blue colour represents negative expansion dominated by Zerodur components and the red color represents positive expansion where Zerodur components are too weak to hold the resonator's expansion. The green zone shows zero expansion where Zerodur contraction and MgF_2 expansion compensate each other. The thermal-expansion coefficient of the crystalline MgF_2 is approximately 9 ppm K^{-1} in the direction that is orthogonal to the crystalline/resonator axis. Based on the simulations, we found that the coefficient can be reduced to 0.15 ppm K^{-1} (60 times) by choosing the proper thickness of the Zerodur layers in the resonator structure described in the inset of Figure 1a unless the stress that propagates to the localized WGM takes into account. We should note here that all other sources of drift such as mechanical creeps or fast gradients of the temperature across the resonator are not eliminated by this technique.

For the compensated resonator, the temperature-dependent resonance frequency shift is introduced by three factors. One of them is the change in the thermorefractive index, which is small (0.6 ppm K^{-1}) for the ordinary axis in MgF_2 crystal. The other two factors are the residual thermal expansion and elasto-optical refractive index changes caused by the stress induced in the material when the resonator expansion is prevented by the Zerodur layers. By using the data for

the thermorefractive effect obtained from the Corning MgF_2 datasheet and data for the elasto-optical effect in ref. [1], we evaluated the stress-induced refractive index change value, averaged over the mode volume and found 0.25-0.5 ppm K^{-1} (the variation is attributed to the uncontrollable changes of the glue layer), which is smaller than the thermorefractive index change (0.6 ppm K^{-1}). The reduction of the impact of the ambient temperature fluctuations on the resonator enables the measurement of the fundamental thermorefractive noise we performed.

The compensation of the free standing resonator may be affected by its mounting technique in a particular resonator's housing. To make sure that our resonator is still compensated after installation, we simulated the expansion of components with an entire mechanical model (Supplementary Figure 1b). Although the complete compensation is lost when the resonator is mounted into the housing, the predicted suppression of the resonator's expansion remains 23× - an acceptable value for this experiment.

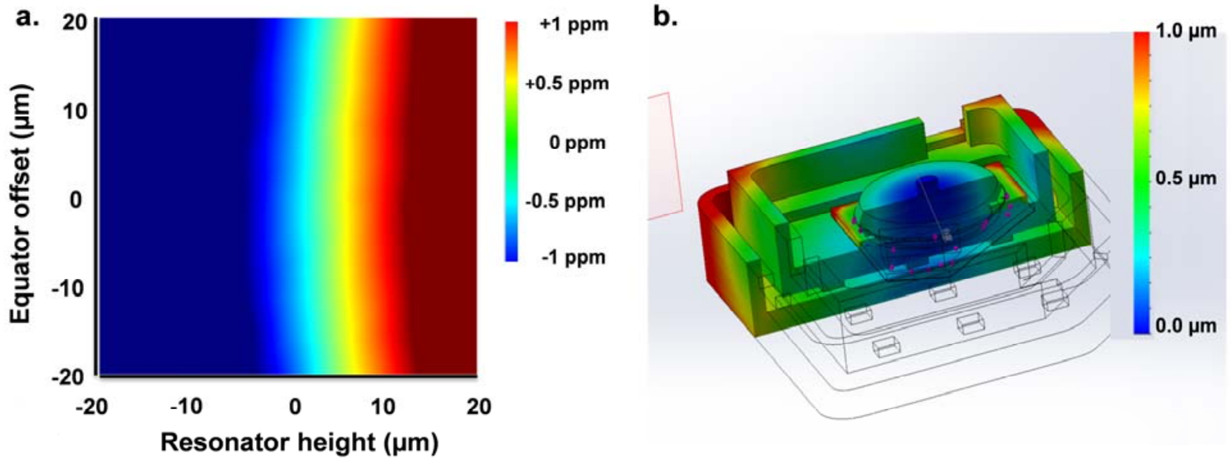

Supplementary Figure 1 | Simulations of the thermal-compensation. a, A simulation of the whispering-gallery-mode (WGM) resonator expansion depending on the device thickness. The colour code indicates the linear expansion of the WGM resonator's equator. The blue colour zone represents negative expansion largely governed by Zerodur components and the red colour zone represents positive expansion where the MgF_2 resonator's expansion is dominant. The green colour zone shows zero expansion where Zerodur contraction and MgF_2 expansion

compensate each other. **b,** A **s**imulation of thermal expansion of the device in the package mount. The colour code indicates the distribution of possible device thermal expansion.

Supplementary Note 2. Thermodynamical noise limits of the cylindrical WGM resonator

The Supplementary Figure 2a illustrates the WGM resonator used in this experiment and its ring-down time (4 μs) shows the loaded Q of 2.4×10^9. The WGM resonator has the radius (r) of 3.45 mm, the rim thickness (L) of 0.025 mm. The thermodynamical noise limit of the compensated MgF_2 WGM resonator is calculated by our theoretical model [2,3]. Here, we consider the two major thermal noise sources that are the thermal-expansion noise and the thermorefractive noise. We numerically quantify the frequency noise power spectral density (FNPSD) imposed by thermal expansion noise for comparing it with experimental measurements using

$$S_\nu^2(f)_{tex} = \nu_0^2 \frac{k_B \alpha_l^2 T^2}{\rho C V_c} \frac{2r^2/\pi^2 D}{1+\left(2fr^2/D\pi\right)^2}, \quad (1)$$

where α_l is the thermal expansion coefficient and V_c is the volume of the WGM resonator. The thermorefractive noise limit is also evaluated by Eq. 2, which is derived for the cylindrical WGM resonator possessing $r >> L$.

$$S_\nu^2(f)_{tre} = \nu_0^2 \frac{k_B \alpha_n^2 T^2}{\rho C V_m} \frac{r^2}{12D}\left[1+\left(\frac{2\pi r^2|f|}{9\sqrt{3}D}\right)^{3/2}+\frac{1}{6}\left(\frac{r^2}{D}\frac{\pi f}{4m^{1/3}}\right)^2\right]^{-1}, \quad (2)$$

where ν_0 is the carrier frequency, k_B is Boltzmann constant, α_n is the thermorefractive coefficient of the material, ρ is the material density, C is the specific heat capacity, V_m is the mode volume, D is the heat diffusion coefficient, and m is the mode order defined by $m=2\pi rn\lambda^{-1}$. This equation shows the $f^{-1.5}$ frequency dependence for the compensated WGM resonator. The values of the parameters used in these simulations are $\alpha_l = 9\times10^{-6}$ K^{-1} (3×10^{-6} K^{-1}) for the conventional (compensated) WGM resonator, $\alpha_n = 6\times10^{-7}$ K^{-1}, T = 300 K, $\rho = 3.18$ g·cm^{-3}, $C = 9.2\times10^6$ erg·g^{-1}·K^{-1}, $V_m = 2.62\times10^{-6}$ cm^3, $D = 7.17\times10^{-2}$ cm^2·s^{-1}, n = 1.37, $V_r = 9.34\times10^{-4}$ cm^3 and λ = 1565.5 nm. We simulate the equations and compare the noise of the thermal-compensation

WGM resonator with that of the conventional WGM resonator as shown in Figure 1b, which shows the thermal compensation reduces the thermal expansion noise below the thermorefractive noise near the carrier frequency. Here, we also experimentally demonstrate the reduction of the thermal expansion noise for the compensated WGM resonator by stabilizing a laser to it. By comparing the FNPSD of the compensated resonator with that of a conventional resonator, possessing approximately equal quality factors, we observe the reduction of frequency noise near the carrier frequency from 1 to 100 Hz by greater than 3 dB as shown in Supplementary Figure 2b.

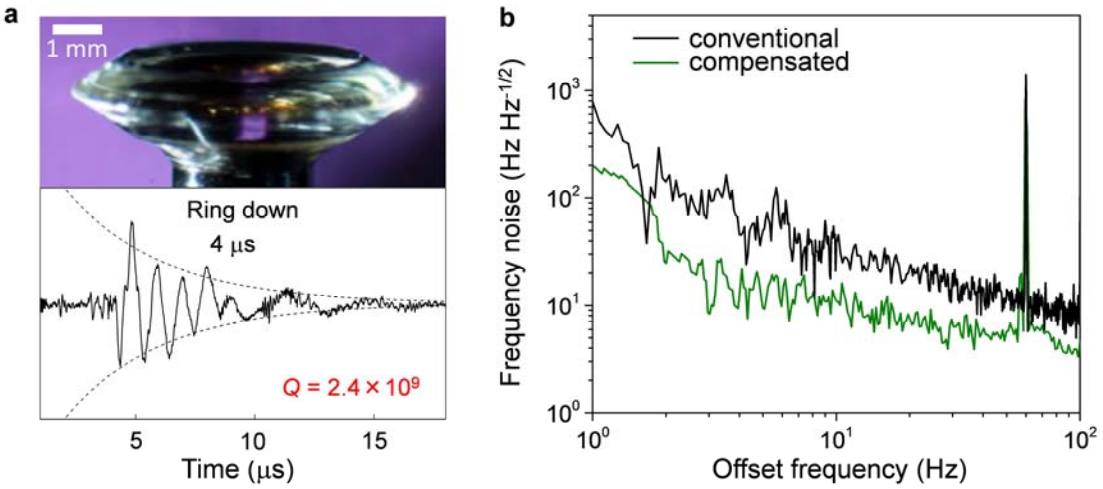

Supplementary Figure 2 | Compensated WGM resonator characteristics. a, The whispering-gallery-mode (WGM) resonator has 6.9 mm in diameter and 0.1 mm in height. The thickness of rim is 25 μm. The ring-down measurement of the resonator shows 4 μs ring-down time corresponding to Q of 2.4×10^9. **b,** Experimentally measured frequency noise of the beat frequency between the WGM resonator-stabilized laser and 1 Hz reference laser for the compensated (olive) and the conventional (black) WGM resonator respectively. The noise is reduced by greater than 3 dB from 1 Hz to 100 Hz offset frequencies.

Supplementary Note 3. Pound-Drever-Hall laser locking and noise characterization

Supplementary Figure 3a illustrates the laser stabilization and noise characterization set-up. A compact 3 kHz linewidth microlaser is coupled into a thermal-compensation WGM resonator in a rigid vacuum chamber and stabilized via Pound-Drever-Hall (PDH) locking technique with both a piezoelectric transducer (PZT) and an acoustic-optical modulator (AOM). The FNPSD and relative frequency instability of the stabilized microlaser are analyzed by heterodyne-beating against a Fabry-Pérot (FP) cavity reference laser, possessing 1 Hz linewidth and less than 0.1 Hz s^{-1} drift-rate. The PDH error signal is optimized for the best laser-WGM resonator stabilization. We modulate our laser frequency by applying a ramping (f_{mod} = 18 Hz) voltage signal to either the piezoelectric transducer (PZT) in the laser or the AOM to search a single WGM resonance near the reference laser frequency (191 THz). The transfer function for laser frequency shift per modulation voltage is 4.5 MHz V^{-1}. Supplementary Figure 3b shows the error signals during the periodic ramping modulation within a half period of time. We carefully optimize the input optical power and polarization to the WGM resonator and phase modulation frequency and amplitude to an electro-optic modulator to find a single error signal (blue curve), which provides tight and stable laser frequency stabilization. Using the weaker error signal from the two resonances (red curve) within the modulation period, the laser frequency stabilization is less stable.

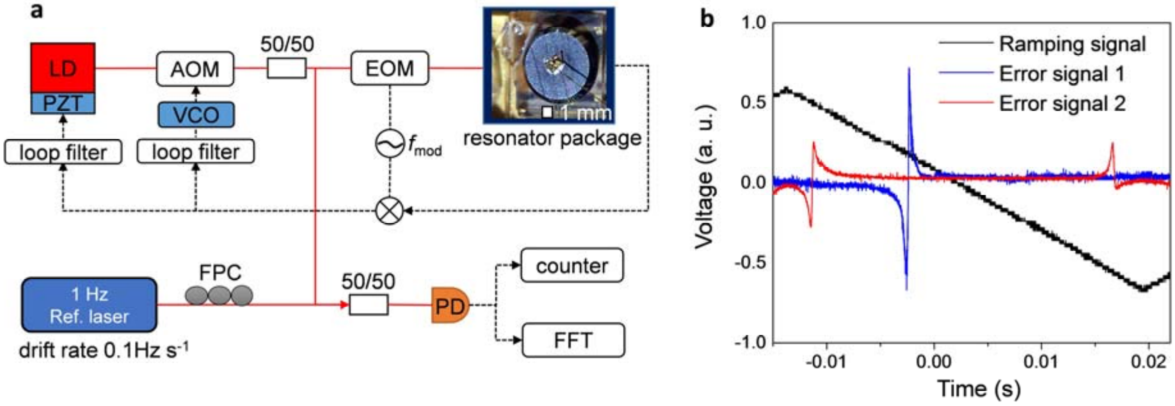

Supplementary Figure 3 | Laser frequency stabilization and noise characterization. a, A microlaser with 3 kHz linewidth is stabilized to the reference whispering-gallery-mode (WGM) resonator via Pound-Drever-Hall (PDH) locking technique. The piezoelectric transducer (PZT) attached onto the microlaser and the external acoustic-optical modulator (AOM) are used to minimize technical noise such that the laser frequency chases a WGM resonance frequency**.** The resonance frequency shift is measured by beating the WGM resonator-stabilized laser frequency against a reference laser possessing 1 Hz linewidth and 0.1 Hz s^{-1} drift rate. LD: laser diode; EOM: electro-optic modulator; PD: photodetector; FPC: fiber polarization controller. **b,** The laser frequency is modulated to search a WGM resonance near the reference laser frequency (191 THz). The ramping modulation voltage with the frequency of 18 Hz is applied to the laser and the laser frequency shift per modulation voltage is 4.5 MHz V^{-1}. The two different error signals corresponding to different resonances are used for stabilizing the laser to the WGM resonator. The tight and stable laser stabilization is only achieved with the strong single error signal (blue curve) within the modulation period.

Supplementary Figure 4a shows the FNPSD curves for two different ambient pressure levels. We observe many noise spikes in the acoustic frequency regime originating from the laboratory environment when the WGM resonator is just sealed without evacuating the vacuum chamber (black curve). These spikes are suppressed by increasing the vacuum level to 1.33 mPa (red curve). The remaining spikes mostly come from the 60 Hz harmonics of the electrical power-line. In this measurement, we observed that the evacuated chamber substantially reduces acoustic noise peaks and provides further noise reduction near the carrier frequency. Since the PZT control loop has a limited feedback bandwidth, we add the second feedback loop using an external AOM to increase the feedback bandwidth for further technical noise suppression. With the AOM feedback loop, the feedback bandwidth is extended over 400 kHz. As a result, the noise over the 20 Hz offset frequencies is far more suppressed than those with only PZT

feedback control (Supplementary Figure 4b), which allows us to investigate the thermodynamical noise limit of the thermal-compensation WGM resonator more clearly.

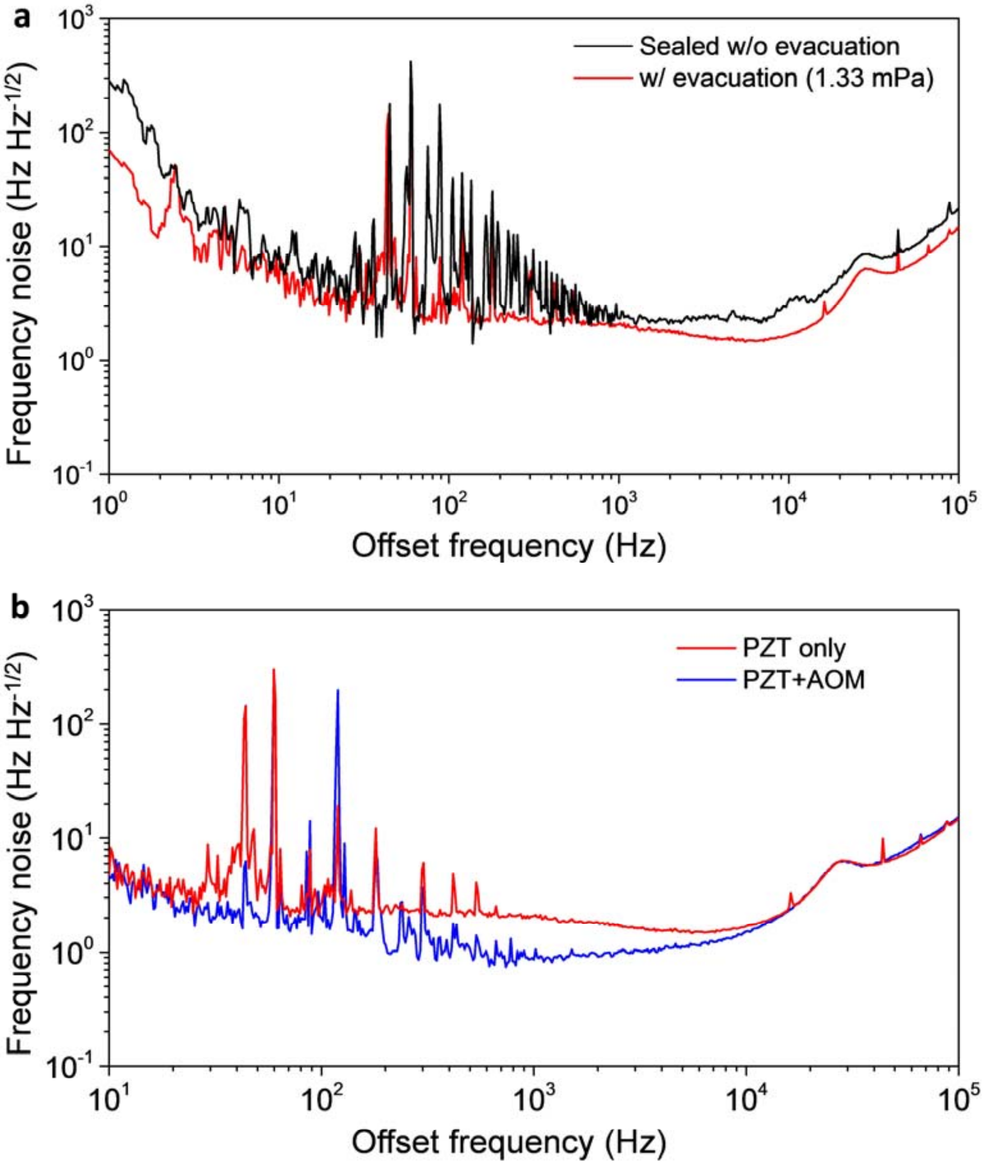

Supplementary Figure 4 | Impacts of the evacuation and the external AOM feedback loop. a, The frequency noise power spectral density (FNPSD) in a sealed chamber without evacuation (black curve). Many spikes are observed in the acoustic noise frequency regime. The FNPSD with evacuation (red curve) in which its pressure is 1.33 mPa. Most of the noise spikes are

mitigated and the remaining spikes are mostly from the 60 Hz harmonics of the electrical power-line. **b,** The FNPSD with PZT only feedback loop (red curve), and the FNPSD with both PZT and acosto-optic modulator (AOM) feedback loops (blue curve). The AOM feedback loop further suppresses noise in the acoustic noise frequency regime. The servo bump at 30 kHz stems from the PZT feedback loop.

Supplementary Note 4. Why thermorefractive noise of a WGM resonator is pink

In our work we reached the fundamental thermodynamical noise floor of the resonator and found out that the power spectrum of the noise scales as $f^{-1.5}$. This is so called pink or flicker noise which is general for various physical processes, ranging from traffic flow to DNA sequence structure [4]. Flicker noise is the major physical mechanism that limits spectral purity of lasers stabilized to super-cavities [5]. The origin of this noise is still not completely understood and is usually studied case-specifically [4-6]. It was theoretically predicted that the fundamental thermorefractive frequency noise of a WGM resonator has distinct pink noise dependence [7]. In this work, we provide a careful experimental study of this noise.

The pink noise in the WGM resonator is a direct consequence of the presence of multiple normal modes of the heat transfer equation in the WGM resonator. The power spectral density of the thermal distribution can be found by summing over these normal modes of a thin resonator of radius R and thickness L (R>>L) [8]

$$S(\Omega)=\frac{k_B T^2}{\rho C V_m}\frac{16D}{R^2}\sum_{p\le \nu^{2/3}}\sum_{m=0,2}\frac{1}{\Omega^2+D^2k_{p,m}^4}\approx\frac{k_B T^2}{\rho C V_m}\frac{R^2}{12D}\left[1+\left(\frac{R^2}{D}\frac{|\Omega|}{9\sqrt{3}}\right)^{3/2}+\frac{1}{6}\left(\frac{R^2}{D}\frac{\Omega}{8\nu^{1/3}}\right)^2\right]^{-1} \qquad (3)$$

where $\Omega=2\pi f$ is the spectral frequency, k_B is the Boltzmann constant, T is the ambient temperature, ρ is the density of the resonator host material, C is the heat capacity of the material, V_m is the volume of the resonator mode, D is the heat diffusion coefficient of the resonator host material, ν is the azimuthal number of the optical WGM, and $k_{p,m}$ is the eigenvector of the heat transfer equation. The power density of the frequency fluctuations of WGMs is proportional to $S(\Omega)$ and its proportionality coefficient is *in situ* the squared thermorefractive coefficient of the

material, α_n^2. From this equation, we find that spectral density of the fundamental thermorefractive noise has Brownian noise frequency dependence (Ω^{-2}) when we only consider one thermal mode of the WGM resonator. However, summing over many modes results in the pink frequency noise ($\Omega^{-1.5}$). Our experimental data confirms this theoretical prediction. This is a fundamentally important observation for a physical structure with limited dimensionality. It is worth noting that similar results were predicted for optical mirror coatings which is important in classical and quantum metrology [8,9].

Supplementary Note 5. Sub-100 Hz linewidth WGM resonator-stabilized laser

Characterizing the spectral linewidth of the cavity-stabilized laser from the FNPSD is not trivial due to the non-unified definition of linewidth of a flickering or drifting laser frequency and therefore we estimate spectral linewidth with two different methods. The integral linewidth is first calculated from the power spectral density with the phase noise method [10]. An effective linewidth of 119 ± 2 Hz is deduced from the raw FNPSD measurement from the olive curve in Figure 3a. We note that most of the residual noise and linewidth contribution arises from the 60 Hz and 120 Hz electrical power line frequency noise. When these two dominant noise peaks are excluded, an upper bound 25 ± 0.3 Hz is estimated. By fitting the frequency noise dependence using frequency decomposition method that removes the rest of the spurious frequencies, we estimate the fundamental noise limited spectral purity and its resultant linewidth is determined to be 8.7 Hz. The spectral linewidth is double-checked by the β-separation line method [11]. This approach implements a simple geometric line based on the low-pass filtered white noise to determine the laser linewidth for an arbitrary frequency noise spectrum. The β-separation line is plotted in Figure 3a (dashed gray line). By integrating the FNPSD up to 16 Hz offset frequency which is the region above the β-separation line, we determine the linewidth of 30.6 ± 0.1 Hz.

Supplementary Note 6. Random walk noise distribution

We calculated the probability distribution [12] for N=100, where N is the number of samples. The probability distributions for the larger N are also similarly calculated. We took 30 sets of

samples of temperature data. Supplementary Table 1 shows the number of upper bound temperature data points (n_u) for each set of data. The average of n_u ($\overline{n_u}$) is calculated by $\frac{1}{30}\sum_{s=1}^{30} n_{us}$ and $\overline{m}$ is derived by $2\overline{n_u} - N$. The p and q are computed by solving the coupled equations that are $p+q=1$ and $p-q=\overline{m}/N$, where p (q) is the probability that the measured temperature point is at the upper (lower) temperature bound. The probability distribution for N=100 is plotted in Figure 5b and its standard deviation, $\overline{(\Delta m)^2}$ is measured by a Gaussian function fit. The standard deviation is approximately $\sqrt{N}$, implying that this measurement follows the random walk distribution.

Set #	1	2	3	4	5	6	7	8	9	10
n_u	37	36	53	52	52	43	53	53	48	63
Set #	11	12	13	14	15	16	17	18	19	20
n_u	37	55	51	11	32	32	37	42	57	77
Set #	21	22	23	24	25	26	27	28	29	30
n_u	47	38	39	75	19	57	53	51	18	40

Supplementary Table 1 | The number of upper bound temperature data points (n_u) for each set of data.

Supplementary References

[1] Szczesniak, J. P. & Corelli, J. C. Stress optical properties of solids in the 1 to 20 micron wavelength region, Rensselaer Polytechnic Institute, Department of Nuclear Engineering, Troy, NY.

[2] Matsko, A. B., Savchenkov, A. A., Yu, N. & Maleki, L. Whispering-gallery-mode resonators as frequency references. I. Fundamental limitations. *J. Opt. Soc. Am. B.* **24**, 1324-1335 (2007).

[3] Savchenkov, A. A., Matsko, A. B., Ilchenko, V. S., Yu, N. & Maleki, L. Whispering-gallery-mode resonators as frequency references. II. Stabilization. *J. Opt. Soc. Am. B.* **24**, 2988-2997 (2007).

[4] Gilden, D. L., Thornton, T. & Mallon, M. W. 1/*f* noise in human cognition. Science **267**, 1837-1839 (1995).

[5] Kessler, T. *et al.* A sub-40-mHz-linewidth laser based on a silicon single-crystal optical cavity. *Nat. Photon.* **6**, 687-692 (2012).

[6] Hooge, F. N., Kleinpenning, T. G. & Vandamme, L. K. Experimental studies on 1/*f* noise. *Reports on progress in Physics* **44**, 480-532 (1981).

[7] Matsko, A. B., Savchenkov, A. A., Yu, N. & Maleki, L. Whispering-gallery-mode resonators as frequency references. I. Fundamental limitations. *J. Opt. Soc. Am. B.* **24**, 1324-1335 (2007).

[8] Braginsky, V. B. & Vyatchanin, S.P. Thermodynamical fluctuations in optical mirror coatings. *Physics Letters A.* **312**, 244-255 (2003).

[9] Evans, M. *et al.* Thermo-optic noise in coated mirrors for high-precision optical measurements. *Phys. Rev. D.* **78**, 102003 (2008).

[10] Hjelme, D. R., Mickelson, A. R. & Beausoleil, R. G. Semiconductor laser stabilized by external optical feedback. *IEEE J. Quantum Electron.* **27**, 352-372 (1991).

[11] Domenico, G. D., Schilt, S. & Thomann P. Simple approach to the relation between laser frequency noise and laser line shape. *App. Opt.* **49**, 4801-4807 (2010).

[12] Reif, F. Fundamentals of statistical and thermal physics. International Edition, chap. **1**, (*McGraw-Hill*, 1985).
